# Posterior Percutaneous Transpedicular Endoscopic Approach for Treating Single-Segment Cervical Myelopathy

**DOI:** 10.1155/2020/1573589

**Published:** 2020-10-22

**Authors:** Ke-Xiao Yu, Wei-Zhong Lu, Chang-Ming Xiao, Lei Chu, Rui Deng, Liang Chen, Zhong-Liang Deng

**Affiliations:** ^1^Department of Orthopaedics, Chongqing Traditional Chinese Medicine Hospital, No. 6 Panxi Seventh Branch Road, Jiangbei District, Chongqing, China; ^2^Department of Spine Surgery, Traditional Chinese Medicine Hospital Affiliated of Southwest Medical University, No. 182, Chunhui Road, Luzhou, Sichuan, China; ^3^Department of Orthopaedics, The Second Affiliated Hospital, Chongqing Medical University, No. 76 Linjiang Road, Yuzhong District, Chongqing, China

## Abstract

**Background:**

Standard posterior percutaneous endoscopic cervical discectomy (PECD) is considered an effective minimally invasive surgery. Although standard PECD can be used to treat radiculopathy with relatively minimal trauma, it is still a challenge to use this approach for treating myelopathy.

**Objective:**

This report is aimed at first describing a posterior transpedicular approach under endoscopy for myelopathy and evaluating the feasibility and short-term clinical effects of this approach.

**Methods:**

In our retrospective analysis between Feb. 2016 to Mar. 2017, 16 patients managed with PECD using the posterior transpedicular approach for symptomatic single-segment myelopathy. Surgery involved drilling 1/2 to 2/3 of the medial portion of the pedicle under endoscopy to provide sufficient space and an appropriate angle for inserting the endoscope into the spinal canal, followed by ventral decompression of the spinal cord. Computed tomography and magnetic resonance imaging were used to evaluate pedicle healing and spinal cord decompression. The primary outcomes included a visual analog scale (VAS) scores of axial neck pain and Japanese Orthopaedic Association (JOA) scores of neurological conditions.

**Results:**

All patients completed a 1-year follow-up examination. The mean duration of surgery was 95.44 ± 19.44 min (52–130 min). The fluoroscopy duration was 5.88 ± 1.05 (4–7). The VAS scores of axial pain significantly improved from 6.94 ± 0.75 preoperatively to 2.88 ± 1.22 postoperatively (*P* < 0.05). The mean JOA scores improved from 8.50 ± 1.12 preoperatively to 14.50 ± 1.46 at the final follow-up (*P* < 0.05). The effects were excellent in 8 cases, good in 6 cases, and fair in 2 cases. After partial pedicle excision, the width of the remaining pedicle was 1.70 ± 0.22 mm postoperatively and significantly recovered to 3.38 ± 0.49 mm at the 1-year follow-up. There were no surgery-related complications, such as dural tearing, spinal cord injury, nerve root injury, pedicle fracture, and cervical hematocele or infection.

**Conclusions:**

The posterior transpedicular approach is an effective method for the treatment of myelopathy in select patients and is a supplement to the described surgical approach for PECD.

## 1. Introduction

Cervical myelopathy is a common degenerative neurological disorder resulting from spinal cord dysfunction, which is generally due to compression by a herniated nucleus pulposus, an osteophyte or a mixture of the two [1, 2]. Surgery is recommended when symptoms deteriorate or conservative treatment fails [2, 3]. Conventional anterior cervical discectomy and fusion (ACDF) is considered the gold standard surgical treatment for single-segment cervical myelopathy, and with advantages such as sufficient decompression and good fusion rates, it is widely applied around the world [4, 5]. However, the loss of segment motion and the expense of implants result in patients and surgeons hesitating to choose this classic modality. When a hard lesion is located in the posterior wall of the vertebral body, even ACDF is invalid, and corpectomy is commonly considered the only effective treatment. In addition, some procedure-related complications have been reported in many studies, including the degeneration of adjacent segments, graft subsidence, pseudarthroses, and access-related complications, which are also concerns [6–8]. Substantial explorations in minimally invasive techniques for avoiding the fusion-related disadvantages and potential disastrous complications of ACDF have been performed.

With an incision length less than 1 cm, percutaneous endoscopic cervical discectomy (PECD) is one of the most minimally invasive methods and has recently become a standard treatment for cervical disc herniation [9, 10]. PECD has shown positive results in the decompression of nerve roots and even provides anterior transcorporeal access for ventral decompression of the spinal cord caused by nucleus pulposus protrusion [11]. However, using existing endoscopic techniques to treat myelopathy caused by osteophytes is challenging. The main problem is the limited operating space in the spinal canal, which is likely to result in iatrogenic nerve root or spinal cord injury. Although the anterior transcorporeal approach can theoretically be used to drill any focal osteophytes while preserving motion and avoiding intervertebral disc violation, pressing the burr on the osteophyte and drilling is very concerning, as during drilling, the force is directed toward the spinal cord, which could easily be injured. Moreover, the tunnel in the vertebral body is too narrow to use an endoscope to directly confirm whether the herniated disc is resected completely or adjust the trajectory to enlarge the decompression range. If a larger operating space could be achieved, all of these problems could be resolved.

In this study, we present a series of cases in which, for the first time, we applied a posterior transpedicular approach under endoscopy. The goals of this newly designed posterior approach are to provide enough space and a sufficient angle to treat cervical myelopathy effectively. We believe that the advantages of this posterior transpedicular approach under endoscopy are safe decompression of the spinal cord under a magnified field, prevention of unnecessary interference with the facet joint, and avoidance of risking injury to significant anterior structures.

## 2. Materials and Methods

### 2.1. Patient Population

A total of 16 patients (10 women, 6 men; mean age, 52 ± 12 years; range, 33–69 years) with cervical myelopathy were treated in our spine center from Feb. 2016 to Mar. 2017. The mean duration of preoperative symptoms was 18.50 ± 10.20 months (range, 5–45 months). Among the patients, 10 patients had myelopathy, and 6 patients had myeloradiculopathy. All surgical procedures were performed by the same surgeon.

The criteria for patient selection for the surgery were as follows: (1) preoperative MRI and CT myelography showing correlations with clinical symptoms and signs; (2) median or paramedian lesion compressing the spinal cord; and (3) myelopathy with or without radiculopathy.

The exclusion criteria were as follows: (1) symptoms caused by dorsal spinal cord compression, such as ligamentum flavum ossification; (2) Pavlov ratio < 0.82 (Pavlov ratio: sagittal spinal canal diameters/sagittal mid-vertebral diameters) or very large osteophytes (the width of the osteophyte exceeds the bilateral margin of the spinal cord in the axial image or exceeds the lower-endplate of the upper vertebrae by more than 0.5 cm); (3) clear segmental instability or deformity; (4) multilevel pathology; (5) previous surgery at the same segment; (6) a suspected infection or tumor in the cervical spine; (7) intolerance of prone position for less than 1.5 h; and (8) inability to provide accurate feedback to the surgeon during the surgery.

### 2.2. Surgical Setup

The main surgical instruments were included a 6.9 mm diameter endoscope with a 25-degree viewing angle, an endoscopic sheath, endoscopic forceps, basket forceps, an endoscopic high-speed diamond burr, a trephine, and a bipolar radiofrequency device.

### 2.3. Surgical Technique

**Figure 1:**
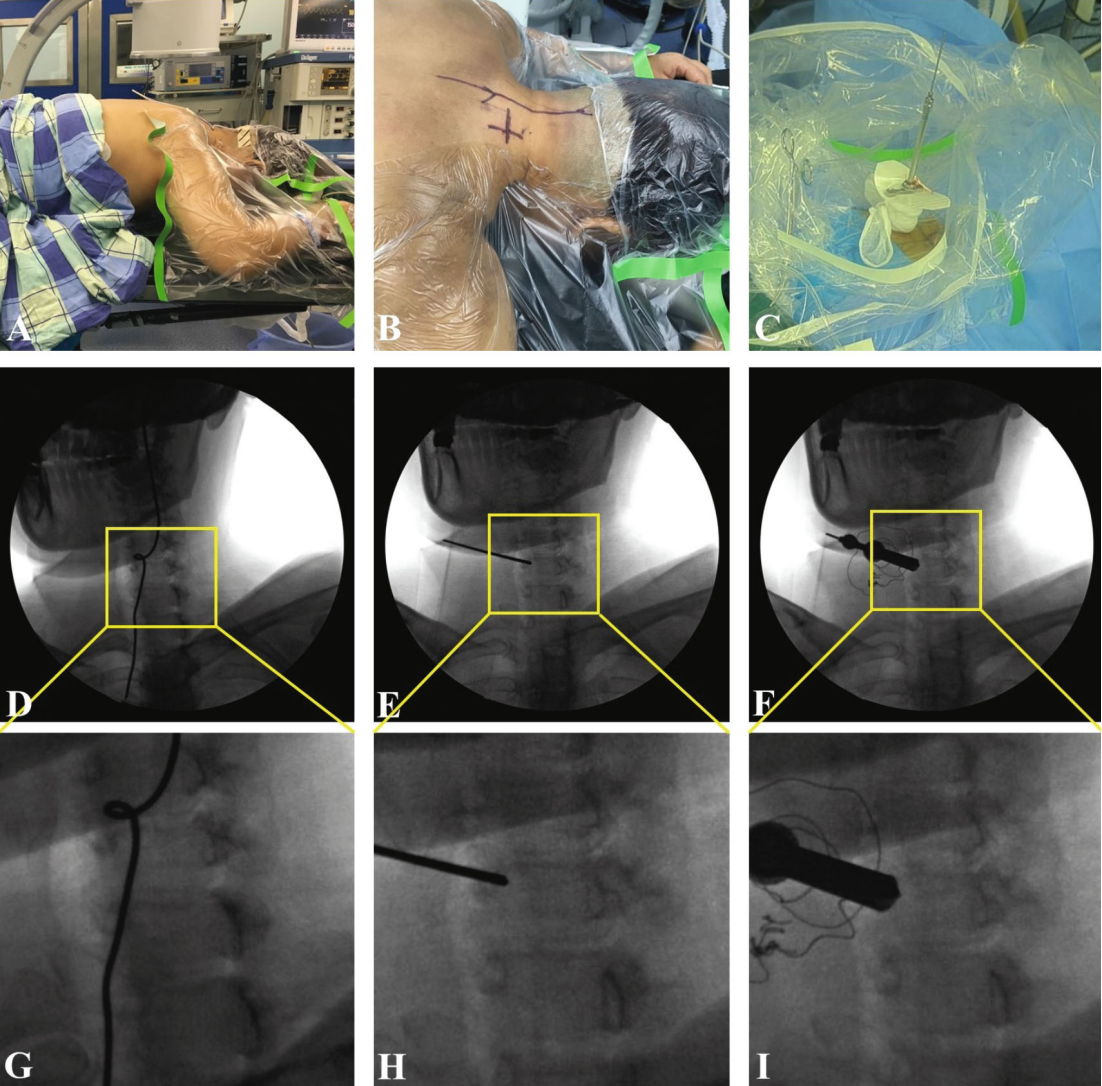
(a) Operative position. The patient is placed in a prone position with the neck in slight flexion. (b) A mark is made on the skin after fluoroscopy. (c) After a dilation sequence along the K-wire, the endoscopic sheath is inserted, and the second-level dilator is replaced by a trephine. (d) The right C6 pedicle is demarcated by 45-degree oblique fluoroscopy and the corresponding local amplification (g). (e) A 2 mm K-wire is inserted through the incision and anchored on the medial margin of the pedicle, as confirmed by 45-degree oblique fluoroscopy and the corresponding local amplification (h). (f) The endoscopic sheath is inserted with the beveled opening facing toward the median and the corresponding local amplification (i).

The patient was placed in the prone position on a radiolucent table with the neck in slight flexion (Figure 1(a)). The head was placed on a silicone head ring and covered with a waterproof membrane, which was fixed in place with tape. No fixation devices, such as the Mayfield system, were necessary.

**Figure 2:**
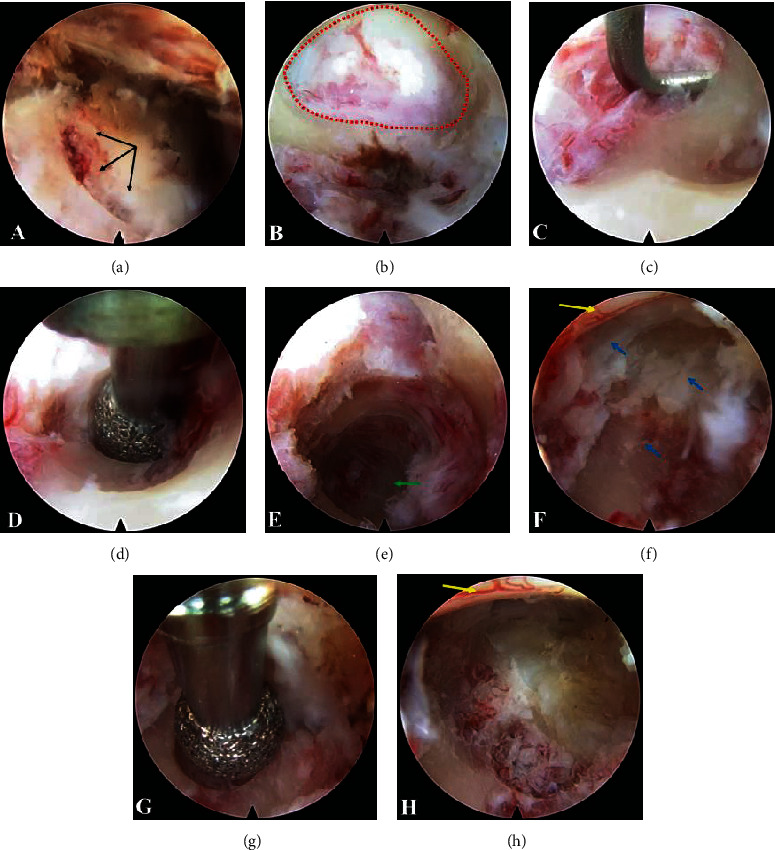
(a) Black arrow indicates the landmark carved by the trephine on the osseous surface of the lateral mass. (b) The red circle indicates the hole drilled at the entry point. (c) A blunt endoscopic hook is used to further dissect the soft tissue and medial margin of the pedicle, which is located lateral to the entry point. (d) The endoscopic high-speed diamond burr is used to drill out the medial portion of the pedicle. (e) After removing the medial portion of the pedicle, the root of the pedicle can be visualized, as indicated by the green arrow. (f) An osteophyte and herniated nucleus pulposus (blue arrow) are located at the axilla of the nerve root and tightly compress the lateral portion of the spinal cord (yellow arrow). (g) The diamond burr is used to remove the osteophyte. (h) Total removal of the herniated disc and osteophyte. The spinal cord (yellow arrow) shows satisfactory decompression.

After the middle part of the target lateral mass (for example, if the lesion located in C5/6 or C6 posterior vertebral wall, the middle part of the ipsilateral lateral mass of C6 was marked) was demarcated by 45-degree oblique fluoroscopy (Figures 1(d) and 2(g)), a mark was made on the skin (Figure 1(b)). In addition to 10 ml for anesthesia of the soft tissue, at least another 5 ml of local anesthetic (lidocaine and bupivacaine at a ratio of 2 : 1) was injected into the bony surface.

**Figure 3:**
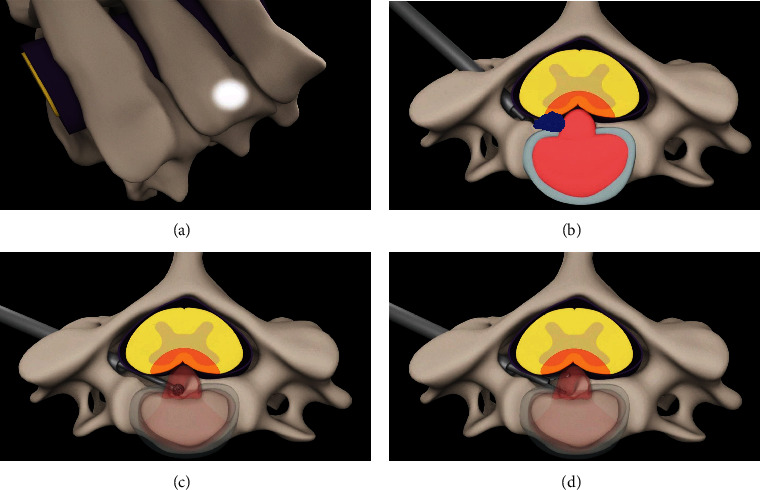
Diagram of the posterior transpedicular approach for PECD. (a) The high bright area located in the lateral mass is the entry point. (b) The medial portion of the pedicle was drilled; then, the diamond burr was used to drill the osteophyte (blue area) located in the posterior wall. (c) Drilling was continued in some of the posterior wall to reach the medial-ventral region of the spinal cord. (d) Forceps were used to grasp the herniated nucleus pulposus.

Taking care to avoid violation of the spinal canal, a 2 mm K-wire was inserted through the incision and anchored on the middle part of the lateral mass, just at the projection of the medial margin of the pedicle, which was confirmed by a 45-degree oblique fluoroscopy (Figures 1(e) and 1(h)). Then, an 8 mm skin incision was made on the mark after local anesthesia was administered. After a sequence of dilation along the K-wire, the endoscopic sheath was inserted with the beveled opening facing the medial (Figures 1(f) and 1(i)). The second dilator was replaced by a trephine, which was rotated clockwise three times on the osseous surface of the lateral mass (Figures 3(a) and 1(c)).

After removing the dilators and trephine, the endoscope was inserted into the endoscopic sheath, and the endoscopic sheath was placed on the osseous surface. Further operation was performed under visual control and continuous irrigation with warm 0.9% saline solution (37°C). After the soft tissue was cleared using forceps and bipolar radiofrequency, the bone surface was exposed, and the landmark carved by the K-wire and trephine was identified (Figure 2(a)). An endoscopic high-speed diamond burr (3 mm in diameter, 17000 r/min) was used to drill a hole at the entry point on the landmark. During the procedure, the upper margin of the lamina and facet joint was kept intact. Once the hole at the entry point was prepared (Figure 2(b)), the deeper soft tissue connecting the cervical spinal canal and lateral mass was identified. A blunt hook was used to further dissect the soft tissue and the medial margin of the pedicle, which was located lateral to the hole at the entry point (Figure 2(c)).

The endoscopic high-speed diamond burr was turned slightly laterally to drill 1/2 to 2/3 of the medial portion of the pedicle forward toward the root of the pedicle along the vertical axis of the pedicle (Figures 2(d) and 2(e)). During the procedure, the lateral margin of the pedicle was kept intact. The endoscope was inserted while the pedicle was being drilled. Low-energy bipolar radiofrequency (less than 15 units) was used to coagulate the venous plexus and soft tissues. The trajectory of the drilling path was always monitored in real-time by endoscopy (Figure 3(b)). After removing the soft tissue and partially protruding nucleus pulposus using endoscopic forceps, the lateral margin of the spinal cord and the axilla of the nerve root were exposed (Figure 2(f)). The diamond burr and forceps were used to continue the drilling forward of the osteophyte and remove the herniated nucleus pulposus (Figures 3(c), 3(d), and 2(g)). After adequate decompression was achieved, as observed through the endoscope, all instruments were removed (Figure 2(h)). The incision was sutured and covered with a water-impermeable dressing. During the entire operation, it is important to maintain communication with the patient to obtain immediate feedback (supplemental video: surgical demonstration animation (available here)).

### 2.4. Postoperative Management

The patient was advised to wear a soft neck collar for 3 weeks. After 3 months, the patient was advised to exercise to strengthen the posterior neck muscles under the guidance of a rehabilitation physician.

### 2.5. Follow-Up

In the clinic, the visual analog scale (VAS) was used to evaluate axial neck pain (neck, nuchal, or shoulder pain), and the Japanese Orthopaedic Association (JOA) score was used to evaluate neurological conditions at 1 day and 1 year postoperatively [12]. The recovery rate was calculated using the following formula: recovery rate (%) = [(postoperative JOA score − preoperative JOA score)/(17–preoperative JOA score)] × 100%, with scores ≥75%, 50% to 74%, 25% to 49%, and <25% considered excellent, good, fair, and poor, respectively [13].

The remaining width of the drilled pedicle and the shortest diameter of the hole at the entry point were measured by CT at 1 day and 1 year postoperatively. During the follow-up examinations at 1 month and 1 year postoperatively, neutral and dynamic cervical radiographs of each patient were obtained. In addition, MRI and CT were performed for a random sample of patients with an excellent or good outcome and for all patients with a fair or poor outcome. These data were collected and measured by a blinded independent observer.

### 2.6. Statistical Analysis

The paired *t*-test was used to compare data obtained preoperatively with data obtained at each follow-up time point. A probability level of less than 0.05 was considered to be the threshold of significance.

## 3. Results

The mean duration of surgery was 95.44 ± 19.44 min (52–130 min), and the mean hospital stay was 5.00 ± 1.62 d (2–10 d). Intraoperative blood loss was negligible. The treatment levels were as follows: C3-4 in 4 patients, C4-5 in 2 patients, C5-6 in 7 patients, and C6-7 in 3 patients. Four patients had herniation-induced compression, five patients had osteophyte-induced compression, and seven patients had compression of a mixed type. The duration of fluoroscopy was 5.88 ± 1.05 (4–7).

There were no surgery-related complications, such as dural tearing, spinal cord injury, nerve root injury, pedicle fracture, and cervical hematocele or infection. A 67-year-old female patient complained of increased postoperative numbness, which may be attributed to long-term (45 months) compression induced by a very large osteophyte. The extensive, high compression on the nerve root significantly reduced the endurance of operative retraction. Finally, the symptoms gradually resolved over 3 months with conservative therapy, and no additional complications have been observed thus far. No patients experienced symptom recurrence during the 1-year follow-up.

All of the patients completed follow-up visits. The mean VAS scores at baseline and 1-day and 1-year postoperatively were 6.94 ± 0.75, 2.88 ± 1.22, and 1.12 ± 0.86, respectively. The VAS score of axial pain showed a significant improvement between before the operation and 1 day after surgery (*P* < 0.05) and further improvement at 1 year postoperatively. The mean JOA scores significantly improved from 8.50 ± 1.12 to 13.68 ± 1.49 immediately after surgery (*P* < 0.05) and further improved to 14.50 ± 1.46 at the 1-year follow-up. The effects were excellent in 8 patients, good in 6 patients, and fair in 2 patients.

**Figure 4:**
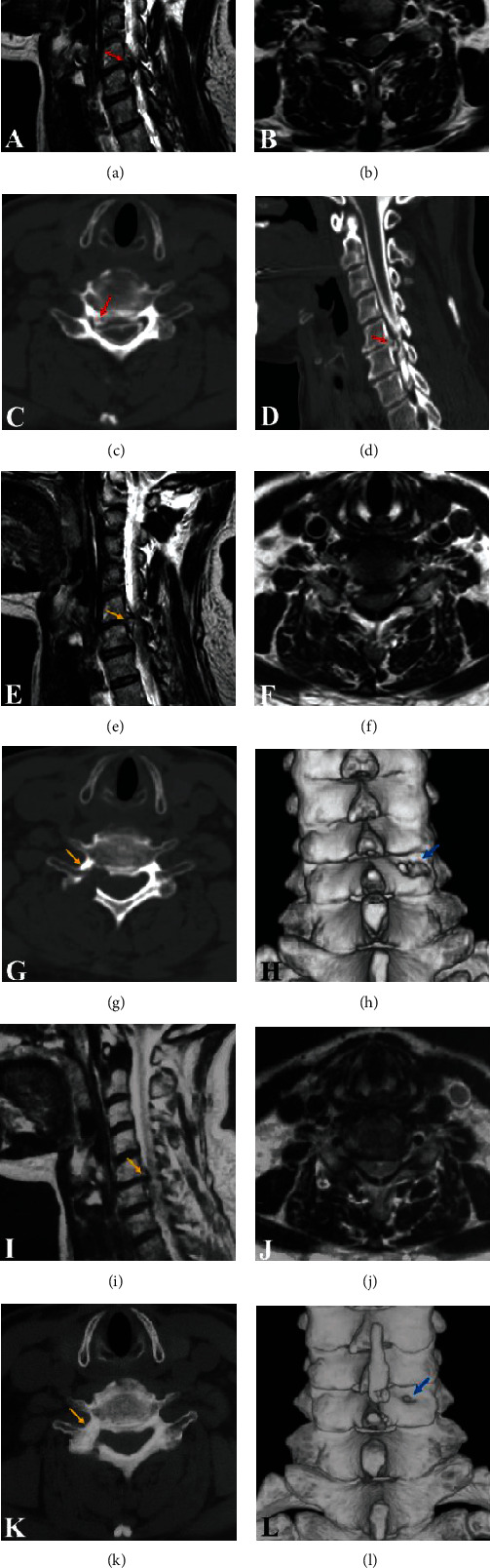
(a) Sagittal MRI scan showing a severe compression of the C5-C6 cervical segment. (b) Axial T2-weighted MRI of the C5-C6 cervical segment. (c) Preoperative CT myelography image showing an osteophyte compressing the lateral portion of the spinal cord. (d) Reconstructed parasagittal CT myelography image showing compression of a mixed type. (e, f) Postoperative sagittal and axial MRI scan showing total decompression. (g) Postoperative CT scan showing partial removal of the lateral mass and pedicle and removal of the osteophyte. (h) 3D reconstruction showing a hole in the lateral mass. Note that the facet joint is intact. (i, j) Postoperative sagittal and axial MRI scans showing no herniated disc at 12 months postoperatively. (k) CT scan at 12 months postoperatively showing that the pedicle and lateral mass defects are obviously decreased. (l) 3D reconstruction showing that the hole in the lateral mass is dramatically decreased compared with its size immediately postoperatively.

**Figure 5:**
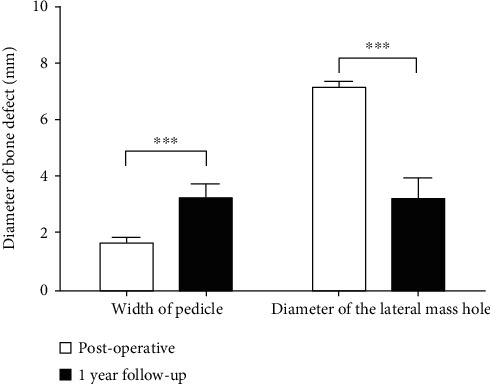
The mean preserved width of the pedicle and the mean shortest diameter of the lateral mass hole immediately postoperatively and at the 1-year follow-up. ^∗∗∗^ indicates a statistically significant difference, *P* < 0.001.

After surgery, adequate decompression of the nerve root was achieved, as determined by MRI and CT (Figures 4(a)–4(g)). At 1 day postoperatively, the medial portion removal of the pedicle (Figure 4(g)) and the hole of the lateral mass were observed on CT, which illustrated that the facet joint was preserved completely (Figure 4(h)). Postoperative sagittal and axial MRI scans showing no herniated disc at 12 months postoperatively (Figures 4(i) and 4(j)). The mean preserved width of the pedicle and the mean shortest diameter of the lateral mass hole showed significant improvement between the 1st day and year postoperatively (1.70 ± 0.22 vs. 3.38 ± 0.49 and 7.07 ± 0.19 vs. 3.08 ± 0.68, respectively, *P* < 0.001, Figure 5). The mean bone defect was significantly decreased, indicating bone healing (Figures 4(g), 4(h), 4(k), and 4 (l)). No patients exhibited cervical instability or increasing kyphosis, as determined by postoperative dynamic radiographic follow-up.

## 4. Discussion

In the era of minimally invasive surgery, the requirements of patients and the goals of surgeons for the treatment of cervical myelopathy or radiculopathy have advanced, promoting the development of therapeutic concepts [14–16]. The conventional strategy of adequate decompression and fusion surgeries, such as the gold standard, ACDF, is challenged by that of nonfusion surgeries, including posterior foraminotomy, microendoscopic foraminotomy, and percutaneous endoscopic discectomy or foraminotomy, which preserve the significant motion of the cervical spine [9, 17–19]. More importantly, in addition to the good therapeutic effect, we believe that minimally invasive surgery not only reduces the incision length but also avoids detrimental effects on vital nongenerative tissues, such as articular cartilage and nervous tissue. These might be the drivers of the evolution of minimally invasive surgery.

With an incision less than 1 cm long, posterior PECD may be considered a minimum invasive surgery performed over the last decade [9, 10, 19–21]. Compared with the gold standard, ACDF, procedures using the “K-hole” technique may provide better exposure to treat compression of the exiting nerve root caused by lateral disc herniation or osteophytes [22, 23]. However, it is still a challenge to treat cervical myelopathy, even for some types of paramedian compression. The main reason is the extremely narrow operating space in the spinal canal. To address this limitation, we temporarily sacrificed 1/2 to 2/3 of the medial portion of the pedicle to provide enough space and a sufficient angle for inserting the endoscope into the spinal canal. Another advantage is the preservation of the facet joint, which reduces the invasiveness of this approach. Although biomechanical research has shown that posterior foraminotomy does not result in cervical segment instability if the facet is resected less than 50% [24], there are still concerns regarding problems related to accelerated postoperative degeneration after facet resection [25, 26]. Therefore, it is important to preserve the facet joint.

During the application of this novel transpedicular approach, there are three key techniques. First, the working channel must be established precisely at the middle of the lateral mass. To confirm the accurate positioning of the drilling entry point, a 2 mm K-wire is used to puncture the projection of the medial-superior margin of the pedicle on the lateral mass under fluoroscopy. Using the anchoring technique, the K-wire is less likely to shift to another location [27]. Based on the anchored K-wire, a trephine can be used to effectively clean the soft tissue, and the circular area can serve as an additional landmark to confirm the drilling entry point. Furthermore, the combination of fluoroscopy and the landmarks carved by the K-wire and trephine provides a coordinate to keep in mind to avoid disorientation in the 2D endoscopic visual field. The anchoring technique is safe and efficacious in the cervical region, as we have demonstrated in C1 vertebroplasty and anterior transcorporeal PECD [11, 28].

The second key technique is the removal of the medial portion of the pedicle. Generally, the endoscope cannot be safely inserted into the spinal canal due to limited space, as insertion may likely result in nerve injury [10]. Therefore, it is difficult to address ventral compression of the nerve root and spinal cord, especially in cases of osteophyte-induced compression. To eliminate the space limitation and allow ventral decompression, we designed the transpedicular approach. After preparing the hole at the entry point, a diamond burr is used to remove the medial half of the pedicle. Based on the accuracy of the position of the hole at the entry point, the drilling procedure is simple. The drilling path follows the cortical bone of the pedicle under endoscopy forward toward the root of the pedicle. In this way, adequate space is provided in the ventral area and around the axilla of the nerve. This space allows the adjustment of the angle of the endoscope and provides a medial trajectory for ventral decompression of the spinal cord. If the compression is more medial, partial removal of the posterior wall can provide additional space for more access to the ventral-medial part of the spinal cord. We preserved the lateral portion of the pedicle with the hope that this bone tissue will heal after surgery, and healing was confirmed by CT at the 1-year follow-up.

The third key technique is hemostasis technology. While the hydraulic pressure of saline irrigation under endoscopy could reduce intraoperative bleeding to help maintain a clear visual field, considerable intraoperative bleeding often occurs from the epidural vessels at the time of epidural dissection or nerve exploration [9]. To address this issue, it is necessary to use radiofrequency to control bleeding. If the bleeding is too severe, it obstructs the visual field, and it becomes challenging to identify the points of bleeding. Currently, we usually try to apply the head of the radiofrequency device to every possible bleeding point: after pressing the head of the radiofrequency to a possible bleeding point, we wait for several seconds; if the field clears, then we can confirm that it was the bleeding point. If the field does not clear, we press the head of the device to other points. This approach with local anesthesia and lower-energy coagulation is less likely to injure nerves.

In China, conventional ACDF results in hospital stays that are usually 7 days; in comparison, the mean hospital stay duration of for PECD using a transpedicular approach was shorter, at 5.00 ± 1.62 d [29]. The average surgical duration was 95.44 ± 19.44 min, which is similar to that of traditional posterior PECD (78.5 min) determined in our previous study. However, in that study, traditional PPECD was used to treat radiculopathy from cervical intervertebral disc herniation, which is much simple than treating myelopathy by removing complex osteophytes [21]. Therefore, the surgical duration of the transpedicular approach of PECD is acceptable. The intraoperative fluoroscopy duration was only 5.88 ± 1.05, which is significantly shorter than 12.6 ± 2.0 in conventional PPECD [27]. These ideal data may contribute to the use of the anchoring technique and the newly designed transpedicular approach. Additionally, the VAS and JOA scores are strong indicators of the excellent therapeutic efficacy.

The concern of this approach is whether it is safe to remove a part of the pedicle. Postoperative bone healing of the drilled pedicle and lateral mass hole was observed on CT. More importantly, the new bone tissue showed formed normally instead of in the form of osteophytes, which may be attributed to Wolff's law and the patients performing proper exercises to strengthen the posterior neck muscles under the guidance of rehabilitation physicians. Postoperative cervical spine instability and increased kyphosis were not found by dynamic radiography during the follow-up; this outcome further demonstrates that this approach is safe and feasible and does not risk iatrogenic injury to the facet joint.

In a newly designed endoscopic cervical transpedicular approach, with obstruction by the spinal cord and nerve root, a straight rod burr cannot drill the osteophytes located in the lower portion of the upper vertebral posterior wall. This problem may be solved with the development of novel endoscopic techniques or surgical instruments. Another disadvantage might be the steep learning curve. For the newly designed endoscopic operative approach, the mean hospital stay and surgical duration were not significantly superior to those of traditional microscopic ACDF, which may be due to the steep learning curve. Although combining fluoroscopy with the carved landmarks increases the convenience of endoscopic localization, it is still important for the operator to be familiar with the endoscopic visual anatomy. Additionally, skillful use of the endoscopic burr and bipolar radiofrequency is necessary. The indications for this transpedicular PECD technique requires further discussion; theoretically, the region of decompression is a restriction. The other limitations of our preliminary research are the lack of randomization and the limited sample size. The transpedicular approach can be used to address compression due to disc herniation and osteophytes; however, it has not been used to treat bilateral compression in a single segment or unilateral compression in multiple segments, as there is a lack of corresponding biomechanical studies. In addition, if the lesions are complex, the combination of many surgical methods may be considered.

## 5. Conclusions

As a supplement to the described surgical approach of PECD, the transpedicular approach is a novel method for establishing access to treat myelopathy in select patients and avoids facet joint damage. The steep learning curve might be the major limitation, and the efficacy and reliability of this approach should be further researched in a large group of patients with a comparative cohort.

## Data Availability

The research related data used to support the findings of this study are restricted by the Ethics Committee of the Second Affiliated Hospital of Chongqing Medical University, in order to protect patient privacy. Data are available from the Corresponding author Zhong-Liang Deng, for researchers who meet the criteria for access to confidential data.

## Supplementary Materials

Supplementary Materials Concise description of the supplementary video: this is an operation demonstration animation, showing the step of transpedicular approach for cervical disc herniation by percutaneous endoscopic technique
